# Supplementary material for: First national tuberculosis patient cost survey in Lao People’s Democratic Republic: Assessment of the financial burden faced by TB-affected households and the comparisons by drug-resistance and HIV status
Source: PLoS One. 2020 Nov 12;15(11):e0241862. doi: 10.1371/journal.pone.0241862 (PMC7660466; doi:10.1371/journal.pone.0241862)
Supplement: S1 Questionnaire — (PDF) [file pone.0241862.s001.pdf]

Lao PDR National TB patient cost survey version TDR 2.0

Part I. Patient information to be obtained from TB treatment card

» Registration of survey participants

DATE OF INTERVIEW *

yyyy-mm-dd

SELECT INTERVIEWER'S NAME *

PATIENT ID NUMBER IN TB TREATMENT CARD *

This should be three digits long in TB treatment card

YEAR OF PATIENT REGISTRATION IN TB TREATMENT REGISTER *

Please answer 2017, 2018 or 2019

NAME OF PROVINCE WHERE THE PATIENT IS REGISTERED *

NAME OF DISTRICT WHERE THE PATIENT IS REGISTERED

CATEGORY OF TREATING FACILITY

Places that stores TB treatment cards

- ◯ Public health centre
- ◯ District hospital
- ◯ Provincial hospital
- ◯ Central, military or police hospital

THE PATIENT'S ID FOR THIS SURVEY IS TDR---.

- ◯ OK

» Patient's general information and TB registration

NAME OF PATIENT

GENDER/SEX *

- ◯ Female
- ◯ Male

AGE (IN COMPLETED YEARS) *

Age must be less than 100 years.

DATE OF SPUTUM SAMPLE COLLECTION

yyyy-mm-dd

BACTERIOLOGICAL AND CLINICAL EXAMINATIONS USED (SEVERAL ANSWERS ARE POSSIBLE)

- ☐ Smear microsocopy
- ☐ Culture
- ☐ Xpert MTB/RIF
- ☐ Chest x-ray
- ☐ Other

SPECIFY "OTHER"

DATE OF DIAGNOSIS

PLACE OF DIAGNOSIS

- ◯ District hospital (higher primary care)
- ◯ Provincial hospital (tertiary care)

SPECIFY "OTHER"

	yyyy-mm-dd	○ Central, military, or police hospital (tertiary care)	
TYPE OF TB<br>○ "Ps+ b+", "Ps+ b-", or "Ps- b+" (bacteriologically confirmed)<br>○ "Ps- b-" (clinically diagnosed)<br>○ EPTB<br>○ Other	SPECIFY "OTHER"	WAS DRUG-SUSCEPTIBILITY TEST (DST) DONE?<br>○ No<br>○ Yes<br>○ Unknown	IF YES, WHAT WAS THE LATEST TEST?<br>○ Gene Xpert MTB/Rif<br>○ Culture with DST
IF YES, WHAT WAS THE RESULTS?<br>○ Rif-resistant<br>○ Non Rif-resistant, Non MDR-TB, but DR-TB<br>○ MDR-TB<br>○ DS-TB<br>○ Unknown	ON MDR-TB TREATMENT? *<br>○ No<br>○ Yes	TREATMENT REGIMEN (DS-TB)<br>○ Standard first line regimen (2HRZE / 4HR)<br>○ Other First line regimen (Miliary TB, meningitis, etc)	TREATMENT REGIMEN (MDR-TB)<br>○ Second-line 9 months' regimen<br>○ Second-line indiviudalized regimen
TOTAL DURATION OF TREATMENT PLANNED (MONTHS)	TREATMENT REGISTRATION GROUP<br>○ 1st line, new<br>○ 1st line, relapse<br>○ 1st line, retreatment after loss to follow-up<br>○ 1st line, retreatment after failure<br>○ Other	SPECIFY "OTHER"	TREATMENT REGISTRATION GROUP<br>○ MDR, new<br>○ MDR, relapse<br>○ MDR, re-treatment after loss to follow-up<br>○ MDR, re-treatment after failure<br>○ Other
SPECIFY "OTHER"	START DATE OF TB TREATMENT *<br>yyyy-mm-dd	THE PATIENT IS CURRENTLY IN THE INTENSIVE OR CONTINUATION TREATMENT PHASE? *<br>○ Intensive phase<br>○ Continuation phase	HAS THE PATIENT COMPLETED HOW MANY DAYS OF THIS PHASE? *
HIV STATUS<br>○ HIV positive<br>○ HIV negative<br>○ Status unknown	ARE YOU CURRENTLY ON TREATMENT FOR ART *<br>○ No<br>○ Yes	ARE YOU CURRENTLY HOSPITALIZED? *<br>○ No<br>○ Yes	IF BEING HOSPITALIZED AT THE TIME OF INTERVIEW, WHEN IS THE PLANNED DATE FOR DISCHARGE?<br>yyyy-mm-dd

CURRENCY USED IN INTERVIEW (DEFAULT IS LAO KIP)

- ◯ KIP
- ◯ Other

THIS PATIENT SHOULD BE EXCLUDED FROM SURVEY UNTIL NEXT VISIT AS HE/SHE IS IN FIRST TWO WEEKS OF THE CURRENT TREATMENT PHASE!

- ◯ OK

Part II. Informed Consent

AT THIS POINT THE INTERVIEWER SHOULD PROVIDE THE PATIENT WITH THE INFORMATION SHEET, EXPLAIN THE STUDY AND WRITTEN INFORMED CONSENT

- ◯ OK

HAS WRITTEN INFORMED CONSENT BEEN OBTAINED OR FINGER-PRINTED? *

- ◯ No
- ◯ Yes

WHY NOT PARTICIPATE?

Select one main reason

- ◯ Language not good enough
- ◯ Time constraints
- ◯ Not comfortable answering questions
- ◯ Other

SPECIFY "OTHER"

DECISION FOR INCLUSION OR EXCLUSION

- ◯ Included
- ◯ Excluded

IF EXCLUDED, REASON FOR EXCLUSION

- ◯ No informed consent
- ◯ Patient within first two weeks of current treatment phase
- ◯ Other

SPECIFY "OTHER"

PERSON WHO IS INTERVIEWEE *

- ◯ Patient
- ◯ DOT supporter/parent/guardian
- ◯ Other

INTERVIEW STYLE *

- ◯ Face-to-face interview
- ◯ Phone interview
- ◯ Other

Part III. Overview of TB treatments before current treatment (for re-treatment cases only)

THIS SECTION IS FOR "OVERVIEW OF TB TREATMENTS BEFORE CURRENT TREATMENT (FOR RE-TREATMENT CASES ONLY)"

- ◯ OK

HOW MANY TIMES HAVE YOU BEEN TREATED FOR TB BEFORE THE CURRENT TREATMENT, INCLUDING COMPLETED AS WELL AS NON-COMPLETED TREATMENTS? *

DO YOU REMEMBER THE FIRST TIME YOU EXPERIENCED TB IN WHICH YEAR?

- ◯ No
- ◯ Yes

WHICH YEAR WERE YOU TREATED FOR TB FOR THE FIRST TIME?

Could you guess if you do not remember exact year?

FOLLOWING QUESTION WILL BE REPEATED TIME(S)

Starting with the oldest treatment

- ◯ OK

» Before*treatment*repeat

Part IV. Costs before the current TB treatment (for new cases in intensive phase only)

THIS SECTION IS FOR "COSTS BEFORE THE CURRENT TB TREATMENT (FOR NEW CASES IN INTENSIVE PHASE ONLY)"

◯ OK

FOR THIS EPISODE OF TB, WHEN DID YOU FIRST EXPERIENCE TB SYMPTOMS? (I.E. HOW MANY WEEKS BEFORE TB TREATMENT STARTED)? *	DID YOU SEEK TREATMENT OR ADVICE FOR SYMPTOMS OF THE CURRENT ILLNESS AT ANY OF THE FOLLOWING TYPES OF HEALTH CARE FACILITIES?	SPECIFY "OTHER"	HOW MANY TIMES DID YOU SEEK CARE OR ADVICE FOR YOUR TB SYMPTOMS ?
Please answer numbers of weeks. If less than a week, answer 1 week<br>*If you did not experience any symptoms, answer 0 week.*	*Before the current TB diagnosis*<br>☐ Village drug kits / Village health worker<br>☐ Public health centre<br>☐ District hospital<br>☐ Provincial hospital<br>☐ Central, military or police hospital<br>☐ Herbalist/traditional healer<br>☐ Private Pharmacy / chemist<br>☐ Private clinic<br>☐ Private hospital<br>☐ No care seeking<br>☐ Other		*Including outpaitent visit and hospitalization*

FOLLOWING QUESTION WILL BE REPEATED TIME(S)
PLEASE ENTER INFORMATION ON FACILITY VISITS IN CHRONOLOGICAL ORDER.

Starting with the oldest visit to health facility

◯ OK

» repeat_before

Part V. Cost during current TB/MDR-TB treatment (to be filled for all patients)

THIS SECTION IS FOR "COST DURING CURRENT TB/MDR-TB TREATMENT (TO BE FILLED FOR ALL PATIENTS)" *

◯ OK

FOLLOWING QUESTIONS ARE RELATED TO "COSTS FOR DOT AND FOOD COSTS DURING AMBULATORY CARE AND COSTS FOR HOSPITALIZATION" *

◯ OK

HAVE YOU BEEN HOSPITALIZED DURING YOUR CURRENT PHASE OF TB TREATMENT DUE TO TB? *

- ◯ No
- ◯ Yes

HOW MANY TIMES WERE YOU ADMITTED/HOSPITALIZED?

FOLLOWING QUESTION WILL BE REPEATED TIME(S) PLEASE ENTER INFORMATION ON HOSPITALIZATIONS IN CHRONOLOGICAL ORDER.

Starting with the oldest hospitalization

- ◯ OK

» -

» Costs for DOTS or drug pick-up

FOLLOWING QUESTIONS ARE RELATED TO "COSTS FOR DOT AND FOOD COSTS DURING AMBULATORY CARE" *

- ◯ OK

ON A DAILY BASIS, DO YOU TAKE YOUR MEDICINES YOURSELF WITHOUT SUPERVISION OR SUPPORT (SELF-ADMINISTERED) OR DO YOU HAVE A TREATMENT SUPERVISOR (DOT) (DIRECT OBSERVED TREATMENT)? *

- ◯ Self administered
- ◯ DOTS

HOW MANY TIMES PER WEEK? *

WHO IS THE DOT (DIRECT OBSERVED TREATMENT) PROVIDER/SUPPORTER THIS TIME?

- ◯ Health provider (health facility)
- ◯ Village health worker/volunteer
- ◯ Family member
- ◯ Friend or relatives
- ◯ Other

SPECIFY "OTHER"

DID YOU TAKE YOUR MEDICINES YOURSELF IN THE INTENSIVE PHASE WITHOUT SUPERVISION OR SUPPORT (SELF-ADMINISTERED) OR DID YOU HAVE A TREATMENT SUPERVISOR OR SUPPORTER (DOT)?

- ◯ Self administered
- ◯ DOTS

WHO WAS THE DOT (DIRECT OBSERVED TREATMENT) PROVIDER/SUPPORTER IN INTENSIVE PHASE?

- ◯ Health provider (health facility)
- ◯ Village health worker/volunteer
- ◯ Family member
- ◯ Friend or relatives
- ◯ Other

SPECIFY "OTHER"

WAS THERE ANY COST FOR THE DOT PROVIDER? *

- ◯ No
- ◯ Yes
- ◯ Unknown

HOW MUCH DID YOU PAY FOR THE LAST VISIT FOR DOT?

HOW LONG DID THE LAST DOT VISIT TAKE, INCLUDING TRAVEL TIME AND WAITING TIME (TOTAL ROUND TRIP TIME)?	IN HOURS	IN MINUTES

WHAT WAS THE COST OF TRANSPORT (ROUND TRIP) FOR THE LAST DOT VISIT, IN TOTAL FOR YOU AND ANY ACCOMPANYING HOUSEHOLD MEMBER?	HOW MUCH DID YOU SPEND ON FOOD AND DRINKS FOR THE LAST DOT (DIRECT OBSERVED TREATMENT) VISIT (ON THE ROAD, WHILE WAITING, LUNCH ETC.), FOR YOU AND ANY ACCOMPANYING HOUSEHOLD MEMBER?

FOLLOWING QUESTIONS ARE RELATED TO "COSTS OF PICKING UP DRUGS AND FOOD COSTS DURING AMBULATORY CARE"

- ◯ OK

DO YOU OR A HOUSEHOLD MEMBER PICK UP TB DRUGS? *	HOW OFTEN DO YOU OR A HOUSEHOLD MEMBER PICK UP TB DRUGS IN THE CURRENT TREATMENT PHASE? *	SPECIFY "OTHER"	WAS THERE A FEE PAID (INCLUDING IN KIND) TO PICK UP DRUGS? (INLUDING FEES PAID BY PATIENT TO COMMUNITY VOLUNTEER OR OTHERS TRAVELLING TO THE FACILITY TO PICKUP DRUGS)
◯ No<br>◯ Yes	◯ Everyday<br>◯ 6 days a week<br>◯ 5 days a week<br>◯ 4 days a week<br>◯ 3 days a week<br>◯ 2 days a week<br>◯ Every week<br>◯ Every two weeks<br>◯ Every month<br>◯ Other		◯ No<br>◯ Yes

HOW MUCH DID YOU PAY FOR THE LAST VISIT FOR DRUG PICK UP? *	WHERE DO YOU OR YOUR HOUSEHOLD MEMBER PICK UP YOUR TB DRUGS?	SPECIFY "OTHER"	WHAT ACCOMMODATION COST DID YOU AND ANY ACCOMPANYING HOUSEHOLD MEMBER HAVE WHEN YOU LAST PICKED UP DRUGS?
	☐ Public health centre<br>☐ District (including military or police) hospital<br>☐ Provincial hospital<br>☐ Central, military, or police hospital<br>☐ Other		

» » Travel and waiting time for drug pick-up

HOW MANY DAYS DID YOU SPEND FOR TRAVEL (ROUND TRIP) TO THE HEALTH FACILITY AND WAITING FOR THE LAST DRUG PICK-UP<br>*If it takes "Days", please answer here in days*<br>........................	HOW MANY HOURS DID YOU SPEND FOR TRAVEL (ROUND TRIP) TO THE HEALTH FACILITY AND WAITING FOR THE LAST DRUG PICK-UP<br>*If it takes "Hours", please answer here in hours*<br>........................	HOW MANY MINUTES DID YOU SPEND FOR TRAVEL (ROUND TRIP) TO THE HEALTH FACILITY AND WAITING FOR THE LAST DRUG PICK-UP<br>*If it takes "Minutes", please answer here in minutes*<br>........................
WHAT WAS THE COST OF TRANSPORT (ROUND TRIP) LAST TIME YOU PICKED UP DRUGS, IN TOTAL FOR YOU AND ANY ACCOMPANYING HOUSEHOLD MEMBER?<br>........................	HOW MUCH DID YOU SPEND ON FOOD AND DRINKS LAST TIME YOU PICKED UP DRUGS (ON THE ROAD, WHILE WAITING, LUNCH ETC.), FOR YOU AND ANY ACCOMPANYING HOUSEHOLD MEMBER?<br>........................	WHAT WAS THE COST OF RECHARGE PHONE CARDS LAST TIME YOU PICKED UP DRUGS FOR YOU AND ANY ACCOMPANYING HOUSEHOLD MEMBERS?<br>........................

FOLLOWING QUESTIONS ARE RELATED TO "COST DURING OUTPATIENT VISITS FOR MEDICAL FOLLOW-UP (SEE THE DOCTOR OR NURSE, HAVE TESTS)" *

◯ OK

HOW MANY TIMES DID YOU HAVE FOLLOW-UP VISITS FOR TB SO FAR DURING THE CURRENT TREATMENT PHASE (TO SEE THE DOCTOR OR NURSE, HAVE FOLLOW-UP TESTS, ETC.)? *<br>........................	HOW MANY TIMES OF FOLLOW-UP VISITS WERE THE SEPARATED VISITS FROM DOTS/DRUG PICK-UP VISITS? *<br>*Among numbers of follow-up visit(s), how many of them were not together with DOT or drug pick-up?*<br>........................

» » Travel and waiting time for follow-up

HOW LONG DID THE LAST FOLLOW-UP MEDICAL OUTPATIENT VISIT TAKE, INCLUDING TRAVEL TIME AND WAITING TIME (TOTAL ROUND TRIP TIME) IN DAYS?<br>*If it takes "Days", please answer here in days*<br>........................	HOW LONG DID THE LAST FOLLOW-UP MEDICAL OUTPATIENT VISIT TAKE, INCLUDING TRAVEL TIME AND WAITING TIME (TOTAL ROUND TRIP TIME) IN HOURS?<br>*If it takes "Hours", please answer here in hours*<br>........................	HOW LONG DID THE LAST FOLLOW-UP MEDICAL OUTPATIENT VISIT TAKE, INCLUDING TRAVEL TIME AND WAITING TIME (TOTAL ROUND TRIP TIME) IN MINUTES?<br>*If it takes "Minutes", please answer here in minutes*<br>........................

WHAT WAS THE COST OF TRANSPORT (ROUND TRIP) AT THE LAST FOLLOW-UP MEDICAL OUTPATIENT VISIT IN TOTAL FOR YOU AND ANY ACCOMPANYING HOUSEHOLD MEMBER?	HOW MUCH DID YOU SPEND ON FOOD AND DRINKS LAST TIME YOU PICKED UP DRUGS (ON THE ROAD, WHILE WAITING, LUNCH ETC.), FOR YOU AND ANY ACCOMPANYING HOUSEHOLD MEMBER?	WHAT ACCOMMODATION COST (OR COSTS ASSOCIATED WITH ACCOMMODATION) DID YOU HAVE FOR THE LAST VISIT, FOR YOU AND ANY ACCOMPANYING HOUSEHOLDMEMBER?	WHAT WAS THE COST OF RECHARGE PHONE CARDS FOR THE LAST VISIT FOR YOU AND ANY ACCOMPANYING HOUSEHOLD MEMBERS? ....
HOW MUCH DID YOU PAY DURING YOUR LAST FOLLOW-UP MEDICAL OUTPATIENT VISIT FOR CONSULTATION?	HOW MUCH DID YOU PAY DURING YOUR LAST FOLLOW-UP MEDICAL OUTPATIENT VISIT FOR RADIOGRAPHY AND OTHER IMAGING?	HOW MUCH DID YOU PAY DURING YOUR LAST FOLLOW-UP MEDICAL OUTPATIENT VISIT FOR TESTS, TB TESTS AND OTHERS?	HOW MUCH DID YOU PAY DURING YOUR LAST FOLLOW-UP MEDICAL OUTPATIENT VISIT FOR OTHER PROCEDURES (FOR BIOPSY, BRONCHIAL LAVAGE ETC. BUT NOT SURGERY UNRELATED TO TB)? ....
HOW MUCH DID YOU PAY AT YOUR LAST FOLLOW-UP MEDICAL OUTPATIENT VISIT FOR TB MEDICINES, INCLUDING PRESCRIPTIONS FOR MEDICINES OUTSIDE HOSPITAL?	HOW MUCH DID YOU PAY DURING YOUR LAST FOLLOW-UP MEDICAL OUTPATIENT VISIT FOR OTHER MEDICINES, INCLUDING NUTRITIONAL SUPPLEMENTS?	WHAT OTHER FEES NOT LISTED IN THE PREVIOUS QUESTIONS DID YOU PAY DURING YOUR LAST FOLLOW-UP MEDICAL OUTPATIENT VISIT (INCLUDING DONATION)?	

COSTS FOR NUTRITIONAL/FOOD SUPPLEMENTS

◯ OK

DO YOU BUY ANY NUTRITIONAL SUPPLEMENTS OUTSIDE YOUR REGULAR DIET BECAUSE OF THE TB ILLNESS, FOR EXAMPLE VITAMINS OR TRADITIONAL MEDICINE? * ◯ No ◯ Yes	IF YES, HOW MUCH DID YOU SPEND ON NUTRITIONAL SUPPLEMENTS IN AN AVERAGE WEEK APPROXIMATELY?	DO YOU BUY ANY FOODS/DRINKS OUTSIDE YOUR REGULAR DIET BECAUSE OF THE TB ILLNESS, FOR EXAMPLE MEAT/FISH, ENERGY DRINKS, OR FRUITS? * ◯ No ◯ Yes	IF YES, HOW MUCH DID YOU SPEND ON FOODS/DRINKS IN AN AVERAGE WEEK APPROXIMATELY? ....

TIME LOSS FOR GUARDIANS
• NOT TO BE FILLED IF THE PATIENT IS UNDER 15 YEARS – FOR CHILDREN, ALL QUESTIONS CONCERNING COSTS, TIME SPENT, INCOME, AND INCOME LOSS IN SECTIONS IV AND V CONCERN COST FOR THE GUARDIAN.
• NOTE: OUT-OF-POCKET COSTS OF TRANSPORT, FOOD, ACCOMMODATION FOR GUARDIAN SHOULD BE INCLUDED IN QUESTIONS ON PART V (TABLES).

◯ OK

DID SOMEBODY IN YOUR HOUSEHOLD ACCOMPANY YOU FOR YOUR LAST DOT VISIT?<br>◯ No<br>◯ Yes	HOW MANY PEOPLE ACCOMPANYING?<br>....................	DID SOMEBODY IN YOUR HOUSEHOLD ACCOMPANY YOU TO PICK UP DRUGS FOR YOUR LAST VISIT?<br>◯ No<br>◯ Yes	HOW MANY PEOPLE ACCOMPANYING?<br>....................
DID SOMEBODY IN YOUR HOUSEHOLD ACCOMPANY YOU FOR YOUR LAST MEDICAL FOLLOW-UP VISIT?<br>◯ No<br>◯ Yes		HOW MANY PEOPLE ACCOMPANYING?<br>....................	DID SOMEBODY IN YOUR HOUSEHOLD ACCOMPANY YOU FOR YOUR LAST HOSPITALIZATION?<br>◯ No<br>◯ Yes
HOW MANY PEOPLE ACCOMPANYING?<br>....................		HOW MANY PERSONS LOST INCOME DURING THAT TIME? (ONLY ACCOMPANYING PEOPLE)<br>....................	

Part VI. Social position and household income

THIS SECTION IS FOR "SOCIAL POSITION AND HOUSEHOLD INCOME"

- OK

DO YOU HAVE ANY OF THE FOLLOWING HEALTH INSURANCE TYPES? *

- None
- National Health Insurance (NHI) scheme
- Community-Based Health Insurance (CBHI)
- Health Equity Fund (HEF)
- Social Security Organization (SSO) for salaried private-sector employees
- State Authority for Social Security (SASS) for civil servants
- Private health insurance
- Other

SPECIFY "OTHER"

WHAT IS YOUR (THE PATIENT'S) EDUCATION LEVEL? *

- Not yet started school
- Not attended school
- Primary
- Lower secondary
- Higher Secondary
- Vocational
- Medium diploma
- Higher diploma/ Bachelor
- Post graduation
- Other

SPECIFY "OTHER"

WHAT IS THE EDUCATION LEVEL OF PRIMARY INCOME EARNER IN THE HOUSEHOLD (IF DIFFERENT FROM PATIENT)?

- Not yet started school
- Not attended school
- Primary
- Lower secondary
- Higher Secondary
- Vocational
- Medium diploma
- Higher diploma/ Bachelor
- Post graduation
- Other

SPECIFY "OTHER"

WHAT IS YOUR MAIN OCCUPATION?

- Unemployed
- Student
- Technician
- Service
- Factory worker
- Farmer
- Government employee (including police, military, teacher)
- Labour
- Retiree
- Homemaker / housewife
- Other

SPECIFY "OTHER"

BEFORE YOU CONTRACTED TB, WHAT WAS YOUR PRIMARY EMPLOYMENT, OR NORMAL WORK, OR NORMAL OTHER MAIN ACTIVITY? *

- Unemployed
- Formal paid work
- Informal paid work
- Retired
- Student
- Housework
- Other

SPECIFY "OTHER"

WHAT IS YOUR PRIMARY EMPLOYMENT, OR NORMAL WORK, OR NORMAL OTHER MAIN ACTIVITY NOW? *

- Unemployed
- Formal paid work
- Informal paid work
- Retired
- Student
- Housework
- Other

SPECIFY "OTHER"

FOLLOWING QUESTIONS ARE RELATED TO "ASSET IN YOUR HOUSEHOLD"

- OK

» Asset Index

IS THE HOUSE YOU ARE STAYING YOUR OWN, FAMILY HOUSE OR RENT? *

- Own
- Family house
- Rent
- Other

WHAT IS YOUR USUAL MAIN SOURCE OF DRINKING WATER? *

- Piped water
- Tube well or borehole
- Dug well
- Water from spring
- Rainwater
- Tanker truck
- Tank
- Surface water
- Bottled water
- Other

WHAT KIND OF TOILET FACILITIES DO YOU OR YOUR HOUSEHOLD HAVE? *

- Piped sewer system
- Septic tank
- Pit latrine
- No toilet facility/ bush/ field (Open defecation)
- Other

WHAT TYPE OF FUEL DOES YOUR HOUSEHOLD MAINLY USE FOR COOKING? (ONE ANSWER ONLY) *

- Electricity
- LPG/natural gas/biogas
- Coal
- Charcoal
- Wood planks
- No food cooked in the household
- Other

» Does your household have following items?

CAR *

- No
- Yes

MOTORBIKE *

- No
- Yes

BICYCLE *

- No
- Yes

HAND TRACTOR *

- No
- Yes

TRICYCLE MOTORBIKE (TUK TUK) *

- No
- Yes

TELEVISION *

- No
- Yes

SATELITE DISK CONNECTION *

- No
- Yes

RADIO *

- No
- Yes

MOBILE PHONE *
- No
- Yes

TELEPHONE *
- No
- Yes

CAMERA *
- No
- Yes

COMPUTER *
- No
- Yes

AIR CONDITIONER/COOL AIR CONDITIONING MACHINE *
- No
- Yes

REFRIGERATOR *
- No
- Yes

SEWING MACHINE *
- No
- Yes

VACUUM CLEANER *
- No
- Yes

WASHING MACHINE *
- No
- Yes

ELECTRIC RICE COOKER *
- No
- Yes

STEAM RICE COOKER *
- No
- Yes

FOOD PROCESSOR *
- No
- Yes

FOLLOWING QUESTIONS ARE RELATED TO "INCOME BEFORE YOU CONTRACTED TB"
- OK

ARE YOU THE PERSON WHO EARNED THE HIGHEST INCOME IN YOUR HOUSEHOLD BEFORE YOU CONTRACTED TB?
- No
- Yes
- Equal contributor

HOW ARE YOU USUALLY PAID (BEFORE YOU CONTRACTED TB)?
- [ ] Bank transferred salary
- [ ] Cash
- [ ] In-kind
- [ ] Cash and in kind
- [ ] Not paid
- [ ] Social welfare benefits
- [ ] Pension scheme
- [ ] Other

SPECIFY "OTHER"

....

HOW MANY HOURS A WEEK WERE YOU WORKING BEFORE YOU CONTRACTED TB?

....

HOW MUCH DO YOU ESTIMATE YOUR AVERAGE INCOME, AFTER TAX, PER MONTH WAS BEFORE YOU CONTRACTED TB? *

Monthly income!

....

HOW MUCH DO YOU ESTIMATE WAS THE AVERAGE MONTHLY INCOME (AFTER TAX) OF YOUR HOUSEHOLD BEFORE YOU CONTRACTED TB? *

(All family member's income must be counted)
Monthly income!

....

FOLLOWING QUESTIONS ARE RELATED TO "CURRENT INCOME AND SOCIAL CONSEQUENCES"
- OK

CURRENTLY, WHAT IS YOUR PRIMARY JOB, OR NORMAL WORK, OR NORMAL OTHER MAIN ACTIVITY?

- [ ] Unemployed
- [ ] Student
- [ ] Technician
- [ ] Service
- [ ] Factory worker
- [ ] Farmer
- [ ] Government employee (including police, military, teacher)
- [ ] Labour
- [ ] Retiree
- [ ] Homemaker / housewife
- [ ] Other

HOW MUCH DO YOU ESTIMATE IS YOUR AVERAGE INCOME, AFTER TAX, PER MONTH AT THE TIME OF TB DIAGNOSIS: ? *

Monthly income!

HOW MUCH DO YOU ESTIMATE IS YOUR HOUSEHOLD AVERAGE INCOME, AFTER TAX, PER MONTH AT THE TIME OF TB DIAGNOSIS: ? *

(All family member's income must be counted)
Monthly income!

HOW MUCH DO YOU ESTIMATE IS YOUR AVERAGE INCOME, AFTER TAX, PER MONTH NOW? *

Monthly income!

NOW, HOW MUCH DO YOU CALCULATE THE AVERAGE INCOME MONTHLY OF YOUR HOUSEHOLD AFTER TAX? *

(All family member's income must be counted)
Monthly income!

NOW, HOW MANY HOURS A WEEK ARE YOU WORKING?

IN THE INTENSIVE PHASE, HOW MANY HOURS DID YOU WORK PER WEEK?

APPROXIMATELY HOW MANY WORKING DAYS OF INCOME HAVE YOU LOST DUE TO YOUR TB ILLNESS OVERALL?

in current treatement phase

DID YOU OR YOUR HOUSEHOLD RECEIVE ANY SOCIAL WELFARE PAYMENT AFTER YOU WERE DIAGNOSED WITH TB? *

- ( ) No
- ( ) Yes

WHAT TYPE OF SOCIAL WELFARE PAYMENT? HOW MUCH DURING THE LAST MONTH?

PAID SICK LEAVE	SOCIAL WELFARE BENEFITS	TB PROGRAM WELFARE SUPPORT	OTHER CASH TRANSFER

HAVE YOU OR A FAMILY MEMBER RECEIVED ASSISTANCE FROM A RELATIVE OR FRIEND NOT LIVING WITH YOU TO COPE WITH TB ILLNESS? *

Not borrowed money

- ◯ No
- ◯ Yes

HOW MUCH DID YOU RECEIVE FROM RELATIVES OR FRIENDS LAST MONTH?

Not borrowed money

CURRENTLY, DO YOU RECEIVE VOUCHERS OR GOODS IN KIND TO COPE WITH TB ILLNESS? *

- ◯ No
- ◯ Yes

WHAT TYPE? HOW MUCH DURING THE LAST MONTH?

TRAVEL VOUCHER

FOOD SUPPORT

OTHER ENABLERS

FROM WHOM DO YOU RECEIVE THE VOUCHER/ GOODS

- ☐ Government
- ☐ NGO
- ☐ Employer
- ☐ Private donation
- ☐ Other

SPECIFY "OTHER"

HOW MANY PEOPLE (ADULTS AND CHILDREN) REGULARLY SLEEP IN YOUR HOUSE (INCLUDING PATIENT)?

Max of 30

HOW MANY ROOMS ARE THERE IN THE HOUSE EXCLUDING THE BATHROOM?

Max of 30

BESIDES YOURSELF, DOES ANYONE ELSE OF YOUR HOUSEHOLD RECEIVE TREATMENT FOR TB, NOW OR DURING THE LAST YEAR? *

- ◯ No
- ◯ Yes

HOW MANY PEOPLE?

HAS THE TB ILLNESS (INCLUDING OTHER HOUSEHOLD MEMBERS TREATED IN THE LAST YEAR) AFFECTED YOUR SOCIAL OR PRIVATE LIFE IN ANY WAY? *

- ☐ No
- ☐ Food insecurity
- ☐ Divorce or separated from spouse/partner
- ☐ Loss of Income (not permanent job loss)
- ☐ Loss of Job
- ☐ Interrupted/dropout/cancel schooling
- ☐ Social exclusion (due to stigma etc)
- ☐ Marriage prospects
- ☐ Other

SPECIFY "OTHER"

Part VII. Coping mechanism

THIS SECTION IS FOR "COPING MECHANISM TO FUND TB RELATED COSTS"<br>◯ OK	DID YOU OR YOUR HOUSEHOLD USE ANY SAVINGS (CASH OR BANK DEPOSITS) TO COVER COSTS DUE TO THE TB ILLNESS? *<br>◯ No<br>◯ Yes<br>◯ No comment - prefer not to answer

» saving_amount

HOW MUCH SAVINGS DID YOU USE?	BEFORE TB TREATMENT STARTED	IN THE INTENSIVE PHASE	IN TOTAL (ONLY IF CANNOT DISAGGREGATE)

HOW MUCH SAVINGS DID YOU USE IN THE CONTINUATION PHASE?	DID YOU BORROW ANY MONEY AS SOLUTIONS TO COVER COSTS DUE TO THE TB ILLNESS? (INCLUDING MORTGAGING ANY ASSETS) *<br>◯ No<br>◯ Yes<br>◯ No comment - prefer not to answer

» Borrow_amount

HOW MUCH DID YOU BORROW?

BEFORE TB TREATMENT STARTED	IN THE INTENSIVE PHASE	IN TOTAL (IF YOU CANNOT DISAGGREGATE)

HOW MUCH DID YOU BORROW IN THE CONTINUATION PHASE?

FROM WHOM DID YOU BORROW?
- ☐ Family and relatives
- ☐ Neighbors/Friends
- ☐ Bank
- ☐ Cooperative (Community Fund)
- ☐ Employer
- ☐ Licensed money lender
- ☐ Unregulated money lender
- ☐ Insurance
- ☐ Other

SPECIFY "OTHER"

HAVE YOU STARTED PAYING BACK THE LOAN?
- ○ No
- ○ Yes

WHEN DID YOU START TO PAY?
- ○ Before treatment started
- ○ During the Intensive phase
- ○ During the continuation phase

WHAT IS THE MONTHLY REPAYMENT ON THE LOAN, INCLUDING INTEREST?

HAVE YOU SOLD ANY OF YOUR ASSETS OR HOUSEHOLD ITEMS TO FINANCE THE COST OF THE TB ILLNESS (INCLUDING PAWNING)? *
- ○ No
- ○ Yes

WHAT DID YOU SELL?
- ☐ Property or land
- ☐ Livestock
- ☐ Transport/vehicle
- ☐ Household item
- ☐ Farm produce or crops
- ☐ Gold/Jewellry
- ☐ Other

SPECIFY "OTHER"

WHEN DID YOU SELL ASSETS OR HOUSEHOLD ITEMS?
- ☐ Before treatment started
- ☐ During the Intensive phase
- ☐ During the continuation phase

HOW MUCH MONEY DID YOU RECEIVE FROM THE SALE OF ALL ITEMS OF YOUR ASSETS OR HOUSEHOLD ITEMS?

BEFORE TB TREATMENT STARTED

IN THE INTENSIVE PHASE

IN TOTAL (IF YOU CANNOT DISAGGREGATE)

HOW MUCH MONEY DID YOU RECEIVE FROM THE SALE OF ALL ITEMS OF YOUR ASSETS OR HOUSEHOLD ITEMS IN THE CONTINUATION PHASE?	THE ASSETS THAT YOU SOLD, WERE THEY PREVIOUSLY SUPPORTING THE FAMILY INCOME (OR EXPENDITURE)? ◯ No ◯ Yes	INDICATE MONTHLY INCOME THAT WAS GENERATED BY THE ASSETS	WHAT IS THE ESTIMATED MARKET VALUE OF THE ASSETS OR HOUSEHOLD ITEMS YOU SOLD?

DID ANYONE IN THE HOUSEHOLD DROP OUT OF SCHOOL OR INTERRUPT SCHOOLING TO ASSIST THE HOUSEHOLD AS A CONSEQUENCE OF YOUR TB ILLNESS? *

- ◯ No
- ◯ Yes

HOW MANY PEOPLE?

FOLLOWING QUESTION WILL BE REPEATED TIME(S)

Based on the number of your family member who dropped out of school as a consequence of your TB

- ◯ OK

» repeat_school dropout

ON A SCALE OF 1 TO 5, IN WHICH 1 IS NO IMPACT AND 5 IS VERY SERIOUS IMPACT, TO WHAT EXTENT HAS THE TB ILLNESS THE FAMILY FINANCIALLY? *

- ◯ 1. No impact
- ◯ 2. Little impact
- ◯ 3. Moderate impact
- ◯ 4. Serious impact
- ◯ 5. Very serious impact

THE IMPACT ON YOUR HOUSEHOLD FINANCIALLY SINCE YOU EXPERIENCED TB SYMPTOMS HAS BEEN THAT YOUR HOUSEHOLD BECAME: *

- ◯ Richer
- ◯ Unchanged
- ◯ Poorer
- ◯ Much poorer

Part VIII. Co-morbidity

THIS SECTION IS FOR "CO-MORBIDITY"

- ◯ OK

DO YOU HAVE ANY OF THE FOLLOWING MEDICAL COMORBIDITIES CURRENTLY? *

Take all diseases that patient has; note all

- ☐ No co-morbidity
- ☐ Type 2 Diabetes Mellitus
- ☐ Chronic Kidney Disease
- ☐ Chronic Obstructive Pulmonary Disease
- ☐ Hypertension
- ☐ HIV
- ☐ Gatro-oesophagel reflux/peptic ulcer disease
- ☐ Hepatitis B or C
- ☐ Depression/anxiety/other mental health
- ☐ Hearing impairement
- ☐ Other

IF YOU DO, WHAT OTHER MEDICAL CONDITIONS DO YOU HAVE?

COMMENTS OF INTERVIEWER
