# Supplementary material for: First national tuberculosis patient cost survey in Lao People’s Democratic Republic: Assessment of the financial burden faced by TB-affected households and the comparisons by drug-resistance and HIV status
Source: PLoS One. 2020 Nov 12;15(11):e0241862. doi: 10.1371/journal.pone.0241862 (PMC7660466; doi:10.1371/journal.pone.0241862)
Supplement: S1 Table — (DOCX) [file pone.0241862.s002.docx]

**S1 Table. List of selected provinces and number of clusters for a tuberculosis patient cost survey in Lao PDR**

| **Province Name** | **No of case in 2017** | **Number of clusters** |
| --- | --- | --- |
| Champasak | 751 | 3 |
| Saisomboun | 11 | 1 |
| Xiengkhuang | 39 | 0 |
| Khammuane | 569 | 2 |
| Phongsaly | 90 | - |
| Oudomxay | 289 | 2 |
| Sekong | 84 | - |
| Vientiane Capital | 1,114 | 5 |
| Attapeu | 160 | - |
| Vientiane Province | 282 | 2 |
| Xaiyabuly | 231 | 1 |
| Luangphabang | 411 | 1 |
| Luangnamtha | 218 | 1 |
| Huaphan | 44 | - |
| Savannakhet | 828 | 4 |
| Saravane | 466 | 2 |
| Bolikhamxay | 152 | 1 |
| Bokeo | 195 | - |
| **Total** | **5,934** | **25** |
