# Supplementary material for: First national tuberculosis patient cost survey in Lao People’s Democratic Republic: Assessment of the financial burden faced by TB-affected households and the comparisons by drug-resistance and HIV status
Source: PLoS One. 2020 Nov 12;15(11):e0241862. doi: 10.1371/journal.pone.0241862 (PMC7660466; doi:10.1371/journal.pone.0241862)
Supplement: S2 Table — (DOCX) [file pone.0241862.s003.docx]

**S2 Table. post-hoc analysis of the incurrence of catastrophic costs by treatment facility**

| Type of treatment facility | Facing catastrophic costs | |
| --- | --- | --- |
|  | Number | % |
| Public health center | 89 | 66.9% |
| District hospital | 244 | 63.9% |
| Provincial hospital | 76 | 55.1% |
| Central, Military, Police hospital | 44 | 61.1% |
| Total | 453 | 62.5% |
