# Supplementary material for: First national tuberculosis patient cost survey in Lao People’s Democratic Republic: Assessment of the financial burden faced by TB-affected households and the comparisons by drug-resistance and HIV status
Source: PLoS One. 2020 Nov 12;15(11):e0241862. doi: 10.1371/journal.pone.0241862 (PMC7660466; doi:10.1371/journal.pone.0241862)
Supplement: S1 Text — (DOCX) [file pone.0241862.s004.docx]

**S1 Text. Types of household assets used for imputing household income and the proportion of participants for whom imputed income had to be employed**

Types of household assets used for imputing household income were car, motor vehicle, bicycle, hand tractor, Tuktuk (auto rickshaw), camera, sewing machine, washing machine, and food processor. For 16 out of 848 (1.9%) survey participants, we used the imputed household income from their asset information.
