# Supplementary material for: First national tuberculosis patient cost survey in Lao People’s Democratic Republic: Assessment of the financial burden faced by TB-affected households and the comparisons by drug-resistance and HIV status
Source: PLoS One. 2020 Nov 12;15(11):e0241862. doi: 10.1371/journal.pone.0241862 (PMC7660466; doi:10.1371/journal.pone.0241862)
Supplement: Attachment — Submitted filename: Review PLOS - AF.docx [file pone.0241862.s005.docx]

Review: PONE-D-20-27870

Introduction.

1. The introduction section is still too long. I suggest authors to shorten this part and directly introduce the Lao, a country that is the focus area of this paper instead of bringing up the Lao part at the end of the intro section.
2. Paragraph 5 (Lines 70-78). This para is more likely the basis for discussion, and is redundant later in discussion part. I suggest to remove this para from intro section and elaborate the data in discussion section.

Methods

3. Study setting. Authors may shorten this part, but explain more on how TB services are delivered in the Lao. What are facilities that deliver the services (public only, or also private; primary care level or only secondary/hospital)? What is the link between facilities and the NTP or NHI? What are cost items covered by the NTP and the NHI; any differences? Self-administered/Directly observed therapy?
4. Sampling (lines 134-135). “..,we enrolled additional and operationally feasible quotas of 120 TB-HIV co-infected patients and 30 DR-TB (i.e. MDR-TB or RR-TB).” What is the basis of this sampling size and quota?
5. Is it not clear if the 25-cluster was randomized from a number of clusters, or it is a total number of clusters in the Lao? How were subjects enrolled (randomly, consecutively, etc)?
6. Inclusion (lines 139-142). Why did authors include children in this study? The TB diagnostic tests for children are different with those for adults and unspecific symptoms and signs occur in many cases. These may lead to a higher cost for diagnosis. They are also more likely to having no job, then we can’t see patients’ financial burden caused in this group. The proportion is very few (0.8%). Is it possible to rule out this group? If it is not possible, authors need to provide a reasonable basis in the method section, analyze the differences between children and adults, and discuss it later in discussion section.

Results

7. The number of DR-TB patients in national representative sample is very few (n=8). We then see some cells are empty (e.g., occupation, insurance type, facility type). In Table 3, only one DR-TB was included in analysis. Are they sufficient number for authors to do inference analysis to see the difference between DS-TB and DR-TB? Please consult to statistician regarding this issue: what is the best way to display it fairly.
8. Authors used clustered sampling method. Do authors need to adjust with the sampling method by using random effects and, for example, generalized linear mixed model for analyses? Please consult to statistician regarding this issue.
9. It seems that nutritional supplement played a significant role in costs incurred by patients. Authors need to define ‘nutritional support’ in methods section or below the table so that readers can easily understand what this term means.
10. Table 4. Why did not authors display costs incurred by guardian(s)? Also, indirect costs in pre-diagnosis phase: are they missed?
11. Table 7. Put ‘Ref’ or ‘1’ in OR column for reference variables.
12. Table 7. Authors analyzed determinants of catastrophic costs for mixed group (DS and DR-TB). The costs in DR-TB group were much higher than the costs incurred by DS-TB group. Why did authors mix the groups in the analyses instead of separate the groups? Since the number of DR-TB subjects are very few, it may be enough to analyze it in descriptive way rather than using regression? Please consult statistician regarding this issue.

Discussion

13. Lines 368: “In Lao PDR, the NTP implements a cash transfer programme for DR-TB patients that provides US$ 5 per day to support expenses for food and transportation.” Was the cash transfer included in costs calculation? Please also confirm it in method section including how the transfer is delivered (condition, unconditional, etc.).

Additional

14. Please avoid using number at the first word in sentence.
15. The paper readability is acceptable, but still need further English language editing.
