# Supplementary material for: First national tuberculosis patient cost survey in Lao People’s Democratic Republic: Assessment of the financial burden faced by TB-affected households and the comparisons by drug-resistance and HIV status
Source: PLoS One. 2020 Nov 12;15(11):e0241862. doi: 10.1371/journal.pone.0241862 (PMC7660466; doi:10.1371/journal.pone.0241862)
Supplement: Attachment — Submitted filename: Response to Reviewers_2_v1.docx [file pone.0241862.s007.docx]

**Response to Reviewers’ Comments:**

Thank you for your positive response to our journal submission. We appreciate the constructive feedback and have strove to incorporate your feedback into our journal article. The itemized responses are as follows:

| **Reviewer 1** | |
| --- | --- |
| 1 | The reasons of using additional sample remains inconsistent and confusing. In lines 111-113, authors wrote that additional respondents were aimed “to assess the difference in the financial burden of TB comparing drug-resistant and drug-susceptible TB patients and for patients with and without TB-HIV co-infection”. However, in lines 156-158, authors stated “Due to different sampling methods for nationally representative sample and additional sample of DR-TB and TB-HIV coinfected patients, statistical tests were performed only in nationally representative sample.” Did authors mean the “assessing the difference” as only in a descriptive way? |
|  | Thank you for your comment. Yes, we aimed at assessing the costs among DR-TB and TB-HIV co-infected patients only in a descriptive way due to the different sampling method and also limited number of samples for DR-TB (even though we enrolled total samples at the time of the survey). |
| 2 | Authors added a group of TB-HIV, but lacked of discussion on this group in discussion section. It is better to discuss the findings in this group if authors thought that adding this group to the sample is really needed. |
|  | Thank you for your suggestion. We added a discussion for TB-HIV as:  Integrated services for TB and HIV was provided only at 11 of 25 central and provincial hospitals, and therefore patients with TB-HIV coinfection had to travel to those hospitals that are usually located far from their residences compared to public health centers or district hospitals, or had to have separate facility visits for TB and HIV treatments [26-28]. Enhancing and decentralizing integrated services for TB and HIV would be necessary to mitigate the financial burden in TB-HIV coinfected patients. |
| 3 | The number of DR-TB patients in national sample were very few and were considerably not enough for statistical comparison. Better if authors mention this shortage in limitation. |
|  | Thank you for the comment. We added following sentences for limitation 2 as:  Furthermore, although we conducted statistical tests comparing various factors between DS-TB and DR-TB patients in the national sample, the number of DR-TB patients in the national sample was very few (N=8) and not enough especially for risk factor analysis for facing catastrophic costs. |
| 4 | Table 3 : Some cells show hours lost in negative values, e.g. -212.2-515. It seems odd, and better to show the values in median (range) instead of mean (95% CI). |
|  | Thank you for your suggestion. We revised table 3 (from mean to median) |
| 5 | Figure 3 is missing. |
|  | We attached all the figures in the submission |
| 6 | Avoid writing numbers at the beginning of a sentence. |
|  | We revised those sentences (mainly in result section). |
